# Association of Guideline Complexity With Individuals’ Ability to Determine Eligibility for COVID-19 Vaccination

**DOI:** 10.1001/jamanetworkopen.2022.34579

**Published:** 2022-10-04

**Authors:** E. Hanna Schurr, Nicole Luisi, Travis Sanchez, Benjamin A. Lopman, Heather Bradley, Patrick S. Sullivan, Aaron J. Siegler

**Affiliations:** 1 Department of Epidemiology, Rollins School of Public Health, Emory University, Atlanta, Georgia

## Abstract

This cross-sectional study examines the association between the complexity of consumer guidelines for COVID-19 vaccination and identification of eligibility.

## Introduction

Initial demand for COVID-19 vaccines exceeded early supply, so states created vaccine prioritization guidance. The US Centers for Disease Control and Prevention also released guidance on prioritization.^1^ We assessed COVID-19 vaccine guideline complexity and the ability of individuals to identify their eligibility.

## Methods

This cross-sectional population-based SARS-CoV-2 serosurvey study used a probability sample of addresses in the US from March 3 to April 21, 2021.^2^ The survey measured demographic factors, COVID-19 vaccine eligibility criteria, and perceived eligibility for vaccination. States a priori selected for this analysis based on having greater than 75 participants were the 5 largest states by population and Georgia (Georgia was oversampled). To determine participant vaccine eligibility, vaccine prioritization guidelines of each state were extracted from government communications (eAppendix 2 in the Supplement). We used survey data to classify participants as vaccine eligible or ineligible based on survey completion date and policy effective date and applied state guidelines to participants’ reports of age, occupation, health conditions, and long-term care facility residence. Individuals were excluded if we could not determine vaccine eligibility (eg, imperfect match between survey-reported occupation and guideline occupation category) or individuals had been vaccinated. Guideline complexity was assessed by total word count and number of eligibility criteria (eAppendix 1 in the Supplement). Sensitivity, specificity, positive predictive value, and negative predictive value were used to compare self-reported perceived vaccine eligibility with correct (study-determined) vaccine eligibility (eTable in the Supplement). The association between guideline complexity and correct vaccine eligibility was determined with adjusted odds ratios (aORs), using logistic regression (SAS, version 9.4; SAS Institute) in which guideline complexity was assessed with states classified as either higher (word count >150, eligibility criteria >30) or lower (word count ≤150, eligibility criteria ≤30) complexity. Comparisons were 2-sided with significance set at α = .05. Emory University Institutional Review Board approved procedures and informed consent process. This study followed the STROBE reporting guideline.

## Results

Of 898 participants, 535 (60%) identified as female; mean age was 45 years (range, 18-89 years). The Figure displays the word count and number of eligibility criteria vs the proportion of participants who correctly identified themselves as eligible. Overall, 72% of participants (310 of 431) correctly indicated their eligibility (sensitivity, 0.72; 95% CI, 0.67-0.76), and 79% of participants (368 of 467) correctly indicated their ineligibility (specificity, 0.79; 95% CI, 0.75- 0.83) (Table). Eligible participants residing in higher complexity states (California, New York, Pennsylvania) were less likely to correctly determine eligibility (61% [92 of 151]; 95% CI, 53%-69%) than persons in states with lower complexity (Florida, Georgia, Texas) (78% [218 of 280]; 95% CI, 73%-83%; OR, 0.44; 95% CI, 0.29-0.68). Adjusting for self-reported potential confounders (age, sex, educational level, race and ethnicity, insurance status, and income) did not substantially change the results (aOR, 0.40; 95% CI, 0.24-0.66). Guideline complexity was not associated with correct determination of ineligibility.

**Figure.**
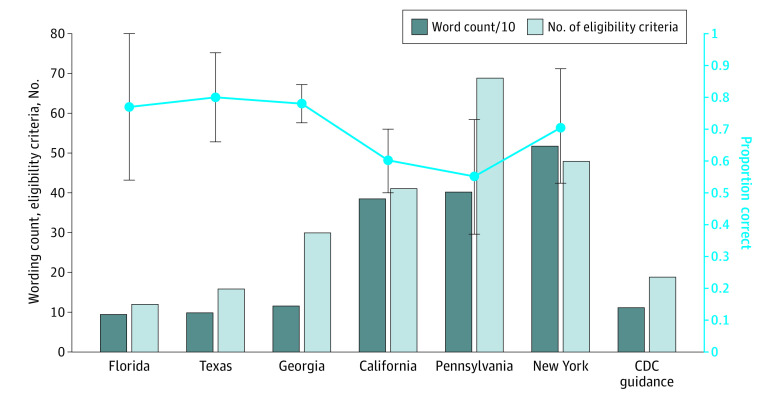
COVID-19 Vaccine Guidelines Complexity and the Proportion of Eligible Persons Correctly Determining Vaccine Eligibility Among a Population-Based Sample of 898 Participants, March-April 2021 Guideline complexity was assessed for each state with total words in the guideline (word count) and with the number of criteria that could make an individual eligible. The proportion of eligible persons correctly determining vaccine eligibility is equivalent to sensitivity (correctly perceived eligible/total actual eligible). For reference, guidance data for guidelines suggested by the US Centers for Disease Control and Prevention (CDC) are displayed, although no states in this study used these exact criteria. Whiskers indicate 95% CIs.

**Table.** Perceived vs Correct Vaccine Eligibility Determination

Catchment area	No.	Sensitivity (95% CI)	Specificity (95% CI)	Predictive value (95% CI)	
				Positive	Negative
All	898	0.72 (0.68-0.76)	0.79 (0.75-0.83)	0.76 (0.70-0.80)	0.75 (0.71-0.79)
Less complex guidelines (Georgia, Texas, Florida)	515	0.78 (0.73-0.83)	0.77 (0.71-0.82)	0.80 (0.75-0.85)	0.74 (0.69-0.80)
More complex guidelines (California, Pennsylvania, New York)	383	0.61 (0.53-0.69)	0.81 (0.76-0.87)	0.68 (0.60-0.76)	0.76 (0.71-0.81)
Georgia	418	0.78 (0.72-0.83)	0.77 (0.71-0.83)	0.82 (0.77-0.87)	0.73 (0.66-0.79)
Texas	46	0.80 (0.66-0.94)	0.69 (0.46-0.91)	0.83 (0.69-0.97)	0.65 (0.42-0.87)
Florida	51	0.77 (0.54-1.00)	0.76 (0.63-0.90)	0.52 (0.30-0.75)	0.91 (0.81-1.00)
California	295	0.60 (0.50-0.70)	0.82 (0.77-0.87)	0.61 (0.51-0.71)	0.81 (0.76-0.87)
Pennsylvania	39	0.55 (0.37-0.73)	0.70 (0.42-0.98)	0.82 (0.68-1.00)	0.35 (0.14-0.56)
New York	49	0.71 (0.55-0.88)	0.76 (0.58-0.94)	0.80 (0.64-0.96)	0.67 (0.48-0.86)

## Discussion

In this study, higher guideline complexity was negatively associated with correct identification of COVID-19 vaccine eligibility during vaccine scarcity in the US. When developing guidance, health agencies must balance precision and clarity. Increased precision may lead to greater complexity and lower target audience comprehension. Our findings suggest potentially large public health implications for complex guidelines. This study is limited owing to ecological design, social desirability bias, and potential misclassification of vaccine eligibility owing to self-reported data.

More complex vaccine guidelines were associated with lower participant comprehension, potentially hindering eligible persons from seeking vaccines during a period of scarcity. To optimize public health communication, brevity and simplicity should not be undervalued.

## Notes

Supplement. **eTable.** Sensitivity and Specificity Calculations **eAppendix 1.** Example of Vaccine Eligibility Criteria Matching **eAppendix 2.** Vaccine Guideline Communication Sources
