# Supplementary material for: Association of Guideline Complexity With Individuals’ Ability to Determine Eligibility for COVID-19 Vaccination
Source: JAMA Netw Open. 2022 Oct 4;5(10):e2234579. doi: 10.1001/jamanetworkopen.2022.34579 (PMC9533177; doi:10.1001/jamanetworkopen.2022.34579)
Supplement: Supplement. — eTable. Sensitivity and Specificity Calculations eAppendix 1. Example of Vaccine Eligibility Criteria Matching eAppendix 2. Vaccine Guideline Communication Sources [file jamanetwopen-e2234579-s001.pdf]

Supplementary Online Content

Schurr EH, Luisi N, Sanchez T, et al. Association of guideline complexity with individuals' ability to determine eligibility for COVID-19 vaccination. *JAMA Netw Open*. 2022;5(10):e2234579. doi:10.1001/jamanetworkopen.2022.34579

eTable. Sensitivity and Specificity Calculations

eAppendix 1. Example of Vaccine Eligibility Criteria Matching

eAppendix 2. Vaccine Guideline Communication Sources

This supplementary material has been provided by the authors to give readers additional information about their work.

© 2022 Schurr EH et al. *JAMA Network Open*.

eTable. Sensitivity and Specificity Calculations

Sensitivity and Specificity for Perceived Eligibility versus Actual Eligibility for COVID-19 Vaccines at time of survey

	**Perceived Eligible**	**Perceived ineligible**	**Total (actual)**
**Actual Eligible**	a	b	a+b
**Actual Ineligible**	c	d	c+d
**Total (Perceived)**	a+c	b+d	

Sensitivity= Correctly perceived eligible /Total actual eligible = a/(a+b)

Specificity= Correctly perceived ineligible /Total actual ineligible= d/(c+d)

Positive Predictive Value= Correctly perceived eligible /Total perceived eligible= a/(a+c)

Negative Predictive Value= Correctly perceived ineligible /Total perceived ineligible= d/(b+d)

© 2022 Schurr EH et al. *JAMA Network Open.*

eAppendix 1. Example of Vaccine Eligibility Criteria Matching
Completed for all states

Date Effective: Date vaccination guideline went into effect
Eligibility Criteria: Criteria under which an individual would be eligible for a COVID-19 vaccine **Citation:** Location of government communication that outlines eligibility criteria
COVIDVu Category? (y/n): If eligibility criteria appear in COVIDVu survey categories (health condition, occupation, age, weight, smoking status)

Texas

Date Effective	Eligibility Criteria	Citation	COVIDVu Category? (y/n)
14-Dec-20	**Frontline Healthcare Personnel (physicians, nurses, EMS providers, home health care workers, emergency response staff, etc)**	COVID-19 Vaccine Information. (n.d.). COVID-19 Vaccine Information. Retrieved September 15, 2021, from https://dshs.texas.gov/covidvaccine/#timeline, Texas Department of State Health Services. (n.d.). COVID-19 Vaccine Allocation Phase 1A Definition [Press release]. https://dshs.texas.gov/coronavirus/immunize/vaccine/EVAP-Phase1A.pdf	Yes
14-Dec-20	**Residents of long-term care facilities**	COVID-19 Vaccine Information. (n.d.). COVID-19 Vaccine Information. Retrieved September 15, 2021, from https://dshs.texas.gov/covidvaccine/#timeline, Texas Department of State Health Services. (n.d.). COVID-19 Vaccine Allocation Phase 1A Definition [Press release]. https://dshs.texas.gov/coronavirus/immunize/vaccine/EVAP-Phase1A.pdf	Yes
29-Dec-20	**Adults 65+**	COVID-19 Vaccine Information. (n.d.). COVID-19 Vaccine Information. Retrieved September 15, 2021, from https://dshs.texas.gov/covidvaccine/#timeline	Yes
29-Dec-20	**Ages 16+ with Cancer**	COVID-19 Vaccine Information. (n.d.). COVID-19 Vaccine Information. Retrieved September 15, 2021, from https://dshs.texas.gov/covidvaccine/#timeline, Department of State Health Services. (n.d.). COVID-19 Vaccine Allocation Phase 1B Definition [Press release]. https://dshs.texas.gov/coronavirus/immunize/vaccine/EVAP-Phase1B.pdf	Yes
29-Dec-20	**Ages 16+ with Chronic kidney disease**	COVID-19 Vaccine Information. (n.d.). COVID-19 Vaccine Information. Retrieved September 15, 2021, from https://dshs.texas.gov/covidvaccine/#timeline, Department of State Health Services. (n.d.). COVID-19 Vaccine Allocation Phase 1B Definition [Press release]. https://dshs.texas.gov/coronavirus/immunize/vaccine/EVAP-Phase1B.pdf	Yes
29-Dec-20	**Ages 16+ with COPD**	COVID-19 Vaccine Information. (n.d.). COVID-19 Vaccine Information. Retrieved September 15, 2021, from https://dshs.texas.gov/covidvaccine/#timeline, Department of State Health Services. (n.d.). COVID-19 Vaccine Allocation Phase 1B Definition [Press release]. https://dshs.texas.gov/coronavirus/immunize/vaccine/EVAP-Phase1B.pdf	Yes
29-Dec-20	**Ages 16+ with Heart conditions (I.e. heart failure, coronary artery disease, or cardiomyopathies)**	COVID-19 Vaccine Information. (n.d.). COVID-19 Vaccine Information. Retrieved September 15, 2021, from https://dshs.texas.gov/covidvaccine/#timeline, Department of State Health Services. (n.d.). COVID-19 Vaccine Allocation Phase 1B Definition [Press release]. https://dshs.texas.gov/coronavirus/immunize/vaccine/EVAP-Phase1B.pdf	Yes
29-Dec-20	**Ages 16+ with solid organ transplantation**	COVID-19 Vaccine Information. (n.d.). COVID-19 Vaccine Information. Retrieved September 15, 2021, from https://dshs.texas.gov/covidvaccine/#timeline, Department of State Health Services. (n.d.). COVID-19 Vaccine Allocation Phase 1B Definition [Press release]. https://dshs.texas.gov/coronavirus/immunize/vaccine/EVAP-Phase1B.pdf	Yes
29-Dec-20	**Ages 16+ with obesity and severe obesity (BMI of 30 kg/m2 or higher)**	COVID-19 Vaccine Information. (n.d.). COVID-19 Vaccine Information. Retrieved September 15, 2021, from https://dshs.texas.gov/covidvaccine/#timeline, Department of State Health Services. (n.d.). COVID-19 Vaccine Allocation Phase 1B Definition [Press release]. https://dshs.texas.gov/coronavirus/immunize/vaccine/EVAP-Phase1B.pdf	Yes
29-Dec-20	**Ages 16+ with pregnancy**	COVID-19 Vaccine Information. (n.d.). COVID-19 Vaccine Information. Retrieved September 15, 2021, from https://dshs.texas.gov/covidvaccine/#timeline, Department of State Health Services. (n.d.). COVID-19 Vaccine Allocation Phase 1B Definition [Press release]. https://dshs.texas.gov/coronavirus/immunize/vaccine/EVAP-Phase1B.pdf	Yes
29-Dec-20	**Ages 16+ with Sickle cell disease**	COVID-19 Vaccine Information. (n.d.). COVID-19 Vaccine Information. Retrieved September 15, 2021, from https://dshs.texas.gov/covidvaccine/#timeline, Department of State Health Services. (n.d.). COVID-19 Vaccine Allocation Phase 1B Definition [Press release]. https://dshs.texas.gov/coronavirus/immunize/vaccine/EVAP-Phase1B.pdf	No
29-Dec-20	**Ages 16+ with Type 2 diabetes mellitus**	COVID-19 Vaccine Information. (n.d.). COVID-19 Vaccine Information. Retrieved September 15, 2021, from https://dshs.texas.gov/covidvaccine/#timeline, Department of State Health Services. (n.d.). COVID-19 Vaccine Allocation Phase 1B Definition [Press release]. https://dshs.texas.gov/coronavirus/immunize/vaccine/EVAP-Phase1B.pdf	Yes
3-Mar-21	**School and licensed child care personnel**	Texas Department of State Health Services. (2021, March 3). School and Child Care Personnel Now Eligible To Be Vaccinated [Press release]. https://dshs.texas.gov/news/releases/2021/20210303.aspx	Yes
15-Mar-21	**Adults 50-64**	COVID-19 Vaccine Information. (n.d.). COVID-19 Vaccine Information. Retrieved September 15, 2021, from https://dshs.texas.gov/covidvaccine/#timeline,	Yes
29-Mar-21	**People ages 16+**	Texas Department of State Health Services. (2021b, March 23). Texas to Open COVID-19 Vaccination to All Adults on March 29 [Press release]. https://dshs.texas.gov/news/releases/2021/20210323.aspx, COVID-19 Vaccine Information. (n.d.). COVID-19 Vaccine Information. Retrieved September 15, 2021, from https://dshs.texas.gov/covidvaccine/#timeline	Yes
12-May-21	**People ages 12+**	Texas Department of State Health Services. (2021c, May 12). Adolescents Ages 12 to 15 Years Now Eligible To Be Vaccinated Against COVID-19 [Press release]. https://dshs.texas.gov/news/releases/2021/20210512.aspx, COVID-19 Vaccine Information. (n.d.). COVID-19 Vaccine Information. Retrieved September 15, 2021, from https://dshs.texas.gov/covidvaccine/#timeline	N/A

© 2022 Schurr EH et al. *JAMA Network Open*.

eAppendix 2. Vaccine Guideline Communication Sources

California

1. Updated COVID-19 Vaccine Eligibility Guidelines. (2021, May 12). California Department of Public Health. https://www.cdph.ca.gov/Programs/CID/DCDC/Pages/COVID-19/VaccineAllocationGuidelines.aspx,
2. How CDC Is Making COVID-19 Vaccine Recommendations. (2021, August 30). Centers for Disease Control and Prevention. https://www.cdc.gov/coronavirus/2019-ncov/vaccines/recommendations-process.html
3. https://www.cdph.ca.gov/Programs/CID/DCDC/Pages/COVID-19/CDPH-Allocation-Guidelines-for-COVID-19-Vaccine-During-Phase-1A-Recommendations.aspx?_cldee=cmxlZ2FyZUBjYWxob3NwaXRhbC5vcmc%3d&recipientid=contact-75ce4068a114eb11a813000d3a375a4d-0012b024d02c4038b3b356af92870a8b&esid=bc1946bd-a439-eb11-a813-000d3a3abdcf&TSPD_101_R0=087ed344cfab2000e0ba0859634e2239dbc62b182a71a9a1c64d6f53f8635f2d9fd4ab927c63d97d087922ff4a1430006dc2d6f90d6dcb522e9751b5aab40b23a6cd0e2fcbc4a792aceb8f43ba4ef622d41d7025d2d31e7ca1a6bd36de1164cd
4. Provider Bulletin. (2021, February 12). California Department of Public Health. https://www.cdph.ca.gov/Programs/CID/DCDC/Pages/COVID-19/Provider-Bulletin-2-12-21.aspx

Florida

1. Fla. Exec. Order No. 20-315 (Dec. 23, 2020)
2. Florida Department of Health. (2021, May 13). Latest Vaccine Updates. Florida Department of Health COVID-19 Outbreak. https://floridahealthcovid19.gov/latest-vaccine-updates/

© 2022 Schurr EH et al. *JAMA Network Open*.

Georgia

1. First Doses of COVID Vaccine Arrive in Georgia. (2020, December 14). [Press release]. https://dph.georgia.gov/press-releases/2020-12-14/first-doses-covid-vaccine-arrive-georgia
2. More Georgians to Become Eligible to Receive COVID-19 Vaccine. (2020, December 31). [Press release]. https://dph.georgia.gov/press-releases/2020-12-30/more-georgians-become-eligible-receive-covid-19-vaccine
3. Georgia Department of Public Health. (2021a, March). COVID-19 Vaccination Plan.
4. Georgia Department of Public Health. (2021b, March). Intellectual and Developmental Disabilities and Complex Medical Conditions Defined for COVID Vaccination Eligibility.
5. Georgia Expands COVID Vaccine Eligibility. (2021, March 11). [Press release]. https://dph.georgia.gov/press-releases/2021-03-10/georgia-expands-covid-vaccine-eligibility
6. Georgia Expands COVID Vaccine Eligibility. (2021, March 24). [Press release]. https://dph.georgia.gov/press-releases/2021-03-24/georgia-expands-covid-vaccine-eligibility

New York

1. Governor Cuomo Updates New Yorkers on State's Vaccination Distribution Plan. (2020, December 9). Governor Cuomo Updates New Yorkers on State's Vaccination Distribution Plan. https://www.governor.ny.gov/news/governor-cuomo-updates-new-yorkers-states-vaccination-distribution-plan
2. Governor Cuomo Updates New Yorkers on Vaccination Efforts Across New York. (2021, January 4). Governor Cuomo Updates New Yorkers on Vaccination Efforts Across New York. https://www.governor.ny.gov/news/governor-cuomo-updates-new-yorkers-vaccination-efforts-across-new-york
3. Governor Cuomo Announces Additional New Yorkers, Individuals 75 and Older Can Begin Scheduling with Providers COVID-19 Vaccination Appointments. (2021, January 11). New York State Governor.

© 2022 Schurr EH et al. *JAMA Network Open*.

https://www.governor.ny.gov/news/governor-cuomo-announces-additional-new-yorkers-individuals-75-and-older-can-begin-scheduling

4. Governor Cuomo Announces New Yorkers with Comorbidities and Underlying Conditions Can Make Appointments at State-Run Mass Vaccination Sites Beginning February 14. (2021, February 8). New York State Governor. https://www.governor.ny.gov/news/governor-cuomo-announces-new-yorkers-comorbidities-and-underlying-conditions-can-make
5. Governor Cuomo Announces State to Expand Vaccination Network to Include Sites Designed to Serve 65-plus Population. (2021, February 26). New York State Governor. https://www.governor.ny.gov/news/governor-cuomo-announces-state-expand-vaccination-network-include-sites-designed-serve-65-plus
6. Governor Cuomo Announces New Yorkers 60 Years of Age and Older and Additional Public Facing Essential Workers Will Be Eligible to Receive COVID-19 Vaccine. (2021, March 9). New York State Governor. https://www.governor.ny.gov/news/governor-cuomo-announces-new-yorkers-60-years-age-and-older-and-additional-public-facing
7. Governor Cuomo Announces New Yorkers 50 Years of Age and Older Will Be Eligible to Receive COVID-19 Vaccine. (2021, March 22). New York State Governor. https://www.governor.ny.gov/news/governor-cuomo-announces-new-yorkers-50-years-age-and-older-will-be-eligible-receive-covid-19
8. Statement from Acting Counsel to the Governor Beth Garvey on Expanded Vaccine Eligibility for Incarcerated Populations. (2021, March 29). New York State Governor. https://www.governor.ny.gov/news/statement-acting-counsel-governor-beth-garvey-expanded-vaccine-eligibility-incarcerated
9. Governor Cuomo Announces New Yorkers 30 Years of Age and Older Will Be Eligible to Receive COVID-19 Vaccine. (2021, March 29). New York State Governor. https://www.governor.ny.gov/news/governor-cuomo-announces-new-yorkers-30-years-age-and-older-will-be-eligible-receive-covid-19
10. Governor Cuomo Accepts NYS Clinical Advisory Task Force Recommendation to Immediately Implement Expanded Use Authorization of the Pfizer COVID-19 Vaccine for 12 to 15-Year-Olds. (2021, May 12). New York State Governor.

© 2022 Schurr EH et al. *JAMA Network Open*.

https://www.governor.ny.gov/news/governor-cuomo-accepts-nys-clinical-advisory-task-force-recommendation-immediately-implement

Pennsylvania

1. Pennsylvania Department of Health. (n.d.). COVID-19 Vaccine Distribution. Retrieved September 18, 2021, from https://www.health.pa.gov/topics/disease/coronavirus/Vaccine/Pages/Distribution.aspx
2. https://www.inquirer.com/news/coronavirus-covid-19-pa-first-vaccine-nj-philly-pandemic-upmc-aid-20201214.html
3. https://www.pennlive.com/news/2020/12/first-covid-19-vaccines-administered-in-pennsylvania.html

Texas

1. COVID-19 Vaccine Information. (n.d.). COVID-19 Vaccine Information. Retrieved September 15, 2021, from https://dshs.texas.gov/covidvaccine/#timeline, Texas Department of State Health Services. (n.d.). COVID-19 Vaccine Allocation Phase 1A Definition [Press release]. https://dshs.texas.gov/coronavirus/immunize/vaccine/EVAP-Phase1A.pdf
2. COVID-19 Vaccine Information. (n.d.). COVID-19 Vaccine Information. Retrieved September 15, 2021, from https://dshs.texas.gov/covidvaccine/#timeline, Department of State Health Services. (n.d.). COVID-19 Vaccine Allocation Phase 1B Definition [Press release]. https://dshs.texas.gov/coronavirus/immunize/vaccine/EVAP-Phase1B.pdf
3. Texas Department of State Health Services. (2021, March 3). School and Child Care Personnel Now Eligible To Be Vaccinated [Press release]. https://dshs.texas.gov/news/releases/2021/20210303.aspx
4. COVID-19 Vaccine Information. (n.d.). COVID-19 Vaccine Information. Retrieved September 15, 2021, from https://dshs.texas.gov/covidvaccine/#timeline,

© 2022 Schurr EH et al. *JAMA Network Open*.

5. Texas Department of State Health Services. (2021b, March 23). Texas to Open COVID-19 Vaccination to All Adults on March 29 [Press release]. https://dshs.texas.gov/news/releases/2021/20210323.aspx
6. COVID-19 Vaccine Information. (n.d.). COVID-19 Vaccine Information. Retrieved September 15, 2021, from https://dshs.texas.gov/covidvaccine/#timeline
7. Texas Department of State Health Services. (2021c, May 12). Adolescents Ages 12 to 15 Years Now Eligible To Be Vaccinated Against COVID-19 [Press release]. https://dshs.texas.gov/news/releases/2021/20210512.aspx
8. COVID-19 Vaccine Information. (n.d.). COVID-19 Vaccine Information. Retrieved September 15, 2021, from https://dshs.texas.gov/covidvaccine/#timeline

© 2022 Schurr EH et al. *JAMA Network Open*.
